# Bilateral idiopathic synchronous testicular infarction mimicking malignancy: a case report

**DOI:** 10.1016/j.eucr.2025.103330

**Published:** 2025-12-23

**Authors:** Joshua Bruinsma, Nisanthan Rajathurai, Hugo C. Temperley, Benjamin Mac Curtain, Mikhail Lozinskiy

**Affiliations:** a Department of Urology, Royal Perth Hospital, Perth, Western Australia, Australia; b Australian Catholic University, Canberra, Australian Capital Territory, Australia; c Trinity St James Cancer Institute, Trinity College Dublin, Dublin, Ireland; d Department of Radiology, St James' Hospital, Dublin, Ireland; e Department of Urology, Lenox Hill Hospital, New York, NY, USA; f Northwell Health, Department of Urology, New Hyde Park, NY, USA

**Keywords:** Testis, Infarction, Bilateral, Idiopathic, Ultrasound, Frozen section, Orchidectomy

## Abstract

Synchronous idiopathic bilateral testicular infarction is exceedingly rare and may mimic malignancy on clinical and radiological assessment. We report a 40-year-old male with bilateral avascular testicular lesions suspicious for germ cell tumour. Intraoperative frozen section during unilateral orchidectomy revealed necrosis without malignancy, avoiding unnecessary bilateral surgery. Final histopathology confirmed infarction with no systemic cause identified. This case highlights the diagnostic challenge of bilateral testicular masses and the value of intraoperative pathology in guiding management.

## Highlights

• First reported case of synchronous idiopathic bilateral testicular infarction.
• Bilateral avascular testicular lesions mimicked malignancy on imaging.
• Frozen section prevented unnecessary bilateral orchidectomy.
• No infectious, vascular or thrombotic cause identified.
• Emphasises importance of multidisciplinary decision-making.

## 1 Introduction

Bilateral testicular infarction is a rare clinical entity most reported in the setting of severe epididymo-orchitis, systemic vasculitis such as polyarteritis nodosa, or prothrombotic conditions including sickle cell disease and coagulation disorders.^1^, ^2^, ^3^, ^4^, ^5^ While unilateral idiopathic infarction is occasionally encountered, bilateral involvement is exceedingly uncommon, and even more so when occurring synchronously.^6^, ^7^, ^8^, ^9^ The pathogenesis remains poorly understood, and idiopathic cases present a diagnostic challenge. Moreover, bilateral solid testicular masses on clinical exam and ultrasound typically prompt strong suspicion for malignancy, often leading to definitive surgical management.^10^ We present the first documented case of synchronous idiopathic bilateral testicular infarction mimicking malignancy, where intraoperative frozen section guided management and avoided overtreatment, illustrating the value of multidisciplinary decision-making in ambiguous presentations.

## 2 Case presentation

A 40-year-old male presented to the Emergency Department with a two-month history of bilateral groin pain and scrotal swelling. He had no history of trauma, cryptorchidism, urinary symptoms, sexually transmitted infections, or recent sexual activity. He denied systemic symptoms including fever, weight loss, or night sweats. Past medical history was unremarkable.

**Fig. 1:**
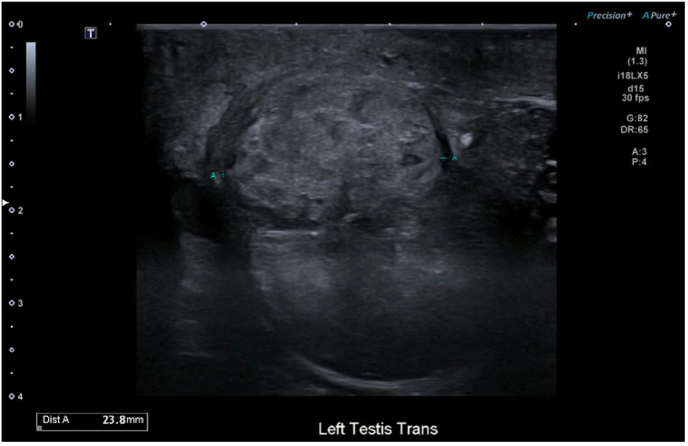
Transverse ultrasound of left testicle.

**Fig. 2:**
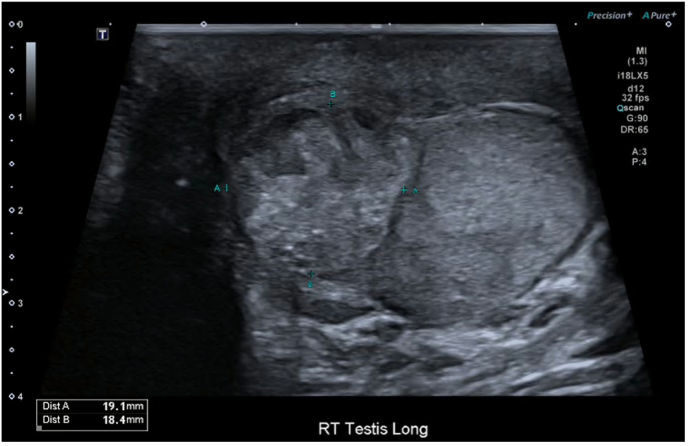
Longitudinal ultrasound of right testicle.

On examination, both testes were enlarged, irregular, and firm, with the left more affected than the right. There was no tenderness or signs of infection. Scrotal ultrasound demonstrated bilateral ill-defined, solid, heterogeneous, avascular testicular lesions measuring 19x19 × 26mm on the right and 24x28 × 19mm on the left (Fig. 1, Fig. 2). Serum tumour markers showed AFP 2, HCG <2, with a mildly elevated LDH 281 (Table 1) which was unable to be explained by prior medical conditions. CT imaging of the chest, abdomen, and pelvis revealed no evidence of metastasis, lymphadenopathy, or infection.

**Table 1:** Tumour markers.

Tumour marker	Value	Reference range
AFP	2 kU/L	<11 kU/L
HCG	<2 IU/L	<2 IU/L
LDH	281 U/L	120–250 U/L

The case was discussed in a multi-disciplinary team meeting with the recommendation of proceeding to bilateral radical inguinal orchidectomy with intra-operative fresh frozen section to exclude haematological malignancy as ultrasound findings were atypical for primary testicular cancer. Percutaneous biopsy of the testicle was considered but not performed due to the theoretical risk of seeding and altering the predictable lymphatic drainage.^11^ A left radical inguinal orchidectomy was performed in a standard manner with intra-operative frozen section. The macroscopic appearance showed areas of necrosis and haemorrhage with microscopy showing extensive necrosis without malignant features. The right sided procedure was abandoned based on the frozen section results. The final histopathology demonstrated altered blood and fibrin on the cut surface macroscopically and diffuse coagulative necrosis of seminiferous tubules with ghost outlines, interstitial haemorrhage, and absent germ cell neoplasia microscopically. There was no evidence of vasculitis, thrombosis, granulomatous inflammation, or malignancy. Special stains for acid-fast bacilli and fungi were negative.

The patient demonstrated an uncomplicated postoperative recovery and was subsequently referred to Rheumatology. Following chart review, Rheumatology deemed a systemic aetiology such as vasculitis to be unlikely, in view of normal inflammatory markers and the absence of histopathological evidence of vasculitis. At six-month follow-up, the patient remained clinically well, reported no features suggestive of hypogonadism and the pain in his remaining testicle had resolved.

## 3 Discussion

Synchronous bilateral testicular infarction is an exceedingly rare clinical entity, with the present case representing what may be the first documented instance of idiopathic, simultaneous involvement of both testes. While unilateral or asynchronous bilateral infarctions have been described in association with vasculitides, infections, or thrombotic disorders, no prior reports were identified describing a truly synchronous idiopathic presentation without identifiable systemic pathology.^1^, ^2^, ^3^, ^4^, ^5^ This case contributes novel insight into the diagnostic challenge of bilateral testicular masses and highlights the critical importance of intraoperative decision-making.

One of the key diagnostic challenges encountered in this case was the radiological mimicry of testicular malignancy. Ultrasound demonstrated bilateral ill-defined, solid, heterogeneous, avascular intratesticular lesions, a pattern that can be seen with germ cell tumours.^12^ Although testicular malignancies more commonly exhibit increased vascularity, this feature may be absent, resulting in lesions being mistakenly interpreted as benign.^13^ Importantly, avascularity does not exclude malignancy with a retrospective study reporting that a substantial proportion of avascular testicular lesions are benign, with only around one-third proving malignant. Notably, lesions with poorly defined margins, as observed in our case, carry a higher likelihood of malignancy, particularly in younger patients or in those with elevated tumour markers or testicular atrophy.^14^

Scrotal magnetic resonance imaging (MRI) was not performed in this case however it represents a potential adjunctive imaging modality in selected patients with equivocal ultrasound findings. Emerging evidence suggests that multiparametric testicular MRI including T2-weighted imaging, diffusion-weighted imaging, and dynamic contrast-enhanced sequences may assist in characterising indeterminate intratesticular lesions and differentiating benign entities such as infarction or haemorrhagic necrosis from germ cell tumours in selected cases.^15^ MRI features such as absent enhancement, low T2 signal intensity, and diffusion restriction patterns have been described in non-viable or infarcted testicular tissue, particularly in the setting of torsion or vascular compromise.^15^^,^^16^ However, MRI availability, cost, and potential delays to definitive management limit its routine use.

A thorough work-up failed to identify any infectious, inflammatory, or prothrombotic cause for the infarction. We propose potential mechanisms include transient low-flow states, subclinical microvascular occlusion, or testicular venous outflow obstruction—possibly exacerbated by the left-sided varicocele observed intraoperatively. However, these remain hypothetical in the absence of definitive histological correlates.

Bilateral testicular lesions present a unique diagnostic challenge, with a broad differential encompassing both benign and malignant aetiologies. Primary bilateral germ cell tumours, though rare, must be considered—particularly seminomas, which can present synchronously and may not always cause elevated tumour markers.^17^ Lymphoma, especially in older men, is another critical consideration, often appearing as ill-defined, hypoechoic, and vascular testicular masses.^18^^,^^19^ Among benign causes, testicular adrenal rest tumours (TARTs) should be recognised in patients with congenital adrenal hyperplasia, particularly those with 21-hydroxylase deficiency, as they can mimic neoplasia and cause infertility.^20^ Granulomatous diseases, including sarcoidosis and tuberculosis, may also manifest with bilateral testicular or epididymal nodularity. Notably, testicular sarcoidosis can present with systemic symptoms and radiological findings indistinguishable from malignancy, such as bilateral testicular nodules and hypercalcaemia.^21^ Infective and inflammatory processes, such as severe epididymo-orchitis and systemic vasculitides like polyarteritis nodosa or IgA vasculitis, can result in bilateral testicular infarction and are typically avascular on imaging.^4^^,^^9^ However, in the absence of infective symptoms in our patient, the clinical likelihood of bilateral infarction was considered low. Burnt-out tumours and metastatic lesions, though uncommon, should also be considered in the appropriate clinical context.

## 4 Conclusion

This case underscores the diagnostic complexity of bilateral testicular lesions, particularly when imaging findings closely mimic malignancy. Synchronous idiopathic bilateral testicular infarction is an exceptionally rare phenomenon, and to our knowledge, this is the first reported case without an identifiable systemic cause. The use of intraoperative frozen section was pivotal in guiding appropriate surgical management and avoiding unnecessary bilateral orchidectomy. This case highlights the importance of a multidisciplinary approach and the need to consider benign mimics of malignancy in atypical presentations.

## CRediT authorship contribution statement

**Joshua Bruinsma:** Writing – review & editing, Writing – original draft, Project administration, Data curation. **Nisanthan Rajathurai:** Writing – review & editing, Conceptualization. **Hugo C. Temperley:** Writing – review & editing. **Benjamin Mac Curtain:** Supervision, Writing – review & editing. **Mikhail Lozinskiy:** Writing – review & editing, Supervision, Conceptualization.

## Funding

This work did not receive funding from any source.

## Conflicts of interest

The authors have no conflict of interest to declare.

## References

[1] Eisner D.J., Goldman S.M., Petronis J., Millmond S.H. (1991). Bilateral testicular infarction caused by epididymitis. AJR Am J Roentgenol.

[2] Ogrodowczyk-Bobik M., Borucka K., Kajdaniuk D. (2021). Bilateral testicular infarction as a complication of acute cardiovascular diseases. Endokrynol Pol.

[3] Ong Lay Keat W., Lechmiannandan S., Manoharan D., Lee S.B., Nagalingam P. (2020). Case report of bilateral testicular infarction due to severe bilateral epididymo-orchitis: a catastrophic complication causing castration. Int J Surg Case Rep.

[4] Stroup S.P., Herrera S.R., Crain D.S. (2007). Bilateral testicular infarction and orchiectomy as a complication of polyarteritis nodosa. Rev Urol.

[5] Toushan M., Atodaria A., Lynch S.D. (2017). Bilateral testicular infarction from IgA vasculitis of the spermatic cords. Case Rep Nephrol.

[6] Huang C.-C., Wen Y.-S. (2007). Idiopathic testicular infarction initially masquerading as urolithiasis and epididymitis. AJEM (Am J Emerg Med).

[7] Fukuhara Y., Shiga Y., Omori Y., Sato K. (2005). [Idiopathic testicular infarction: a case report]. Hinyokika kiyo Acta urologica Japonica.

[8] Fukuda S., Takahashi T., Kumori K. (2008). Idiopathic testicular infarction in a boy initially suspected to have acute epididymo-orchitis associated with mycoplasma infection and Henoch–Schönlein purpura. J Pediatr Urol.

[9] Kurokawa M., Naito S., Ichiyanagi O. (2016). [Asynchronous Bilateral Testicular Infarction with Suspected Polyarteritis Nodosa : A Case Report]. Hinyokika kiyo Acta urologica Japonica.

[10] EAU Guidelines (2025).

[11] Corby H.M., Lynch T.H., Fitzpatrick J.M., Smith J.M. (1996). Inguinal lymph node metastases from a testicular tumour. Br J Urol.

[12] Belfield J., Findlay-Line C. (2022). Testicular germ cell tumours-the role of conventional ultrasound. Cancers (Basel).

[13] Huang D.Y., Sidhu P.S. (2012). Focal testicular lesions: colour doppler ultrasound, contrast-enhanced ultrasound and tissue elastography as adjuvants to the diagnosis. Br J Radiol.

[14] Ma W., Sarasohn D., Zheng J., Vargas H.A., Bach A. (2017). Causes of avascular hypoechoic testicular lesions detected at scrotal ultrasound: can they be considered benign?. AJR Am J Roentgenol.

[15] Saidian A., Bagrodia A. (2023). Imaging techniques to differentiate benign testicular masses from germ cell tumors. Curr Urol Rep.

[16] Watanabe Y., Nagayama M., Okumura A. (2007). MR imaging of testicular torsion: features of testicular hemorrhagic necrosis and clinical outcomes. J Magn Reson Imag.

[17] Liu L., Wang C., Shah S. (2021). Synchronous bilateral primary testicular tumors with discordant histopathology. Cureus.

[18] Trama F., Illiano E., Aveta A., Pandolfo S.D., Bertuzzi G., Costantini E. (2021). Bilateral primary testicular diffuse large B-CELL lymphoma. Urol Case Rep.

[19] Goodman J.D., Carr L., Ostrovsky P.D., Sunshine R., Yeh H.-C., Cohen E.L. (1985). Testicular lymphoma: sonographic findings. Urol Radiol.

[20] Piskinpasa H., Ciftci Dogansen S., Kusku Cabuk F. (2019). Bilateral adrenal and testicular mass in a patient with congenital adrenal hyperplasia. Acta Endocrinol (Buchar).

[21] Handa T., Nagai S., Hamada K. (2003). Sarcoidosis with bilateral epididymal and testicular lesions. Intern Med.

